# Low‐intensity urbanisation alters community composition across multiple trophic levels on Lipsi Island, Greece

**DOI:** 10.1002/ece3.70135

**Published:** 2024-08-06

**Authors:** Eleanor R. Hawkins, Laura Macrina, Alice Malcolm‐McKay, Nicola Cotterill, Anastasia Miliou, Eoin J. O'Gorman

**Affiliations:** ^1^ School of Life Sciences University of Essex Colchester UK; ^2^ Marine Science Program, Division of Biological and Environmental Science and Engineering King Abdullah University of Science and Technology (KAUST) Thuwal Saudi Arabia; ^3^ Archipelagos Institute of Marine Conservation Agios Konstantinos Samos Greece

**Keywords:** butterfly diversity, community composition, urban exploiters, urban greenspace, urbanisation

## Abstract

Urbanisation has reduced the abundance and diversity of many taxonomic groups, and the effects may be more pronounced on islands, which have a smaller regional species pool to compensate. Green spaces within urban environments may help to safeguard wildlife assemblages, and the associated habitat heterogeneity can even increase species diversity. Here, total abundance and species diversity of butterflies, birds, and vegetation at nine rural and nine urban locations were quantified on Lipsi Island, Greece. Sites were assessed using Pollard walks for butterflies, point‐count surveys for birds, and quadrats for vegetation. There was no significant difference in the abundance or species diversity of butterflies or vegetation among rural and urban locations, which could pertain to the low building density within urbanised areas and the minimal extent of urbanisation on the island. However, urban areas hosted a significantly greater abundance, richness, and diversity of birds compared to rural sites. The community composition of butterflies, birds, and vegetation also differed significantly between urban and rural locations, highlighting the impact of urbanisation on species across a broad range of trophic groups. This study contributes to ecological knowledge on the impacts of urbanisation across multiple trophic levels in island ecosystems, with comparisons across a gradient of island size and urbanisation intensity needed in future research.

## Graphical abstract

Urbanisation has reduced the abundance and diversity of many taxonomic groups, and the effects may be more pronounced on islands, which have a smaller regional species pool to compensate. Green spaces within urban environments may help to safeguard wildlife assemblages, and the associated habitat heterogeneity can even increase species diversity. This study contributes to ecological knowledge on the impacts of urbanisation across multiple trophic levels in island ecosystems, with comparisons across a gradient of island size and urbanisation intensity needed in future research.

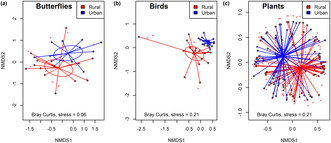

## 1 INTRODUCTION

In the debate regarding global biodiversity decline, urban areas can both be a filter and enhancer of biodiversity, with different factors and context determining the outcomes (Cardinale et al., 2018; Lepczyk et al., 2023; Uchida et al., 2021). Fragments of artificial spaces such as parks, gardens and other green areas may provide a diverse plant composition and fulfil ecosystem functions needed to maintain urban wildlife (Swan et al., 2021; Townsend, 2008), but they also host ornamental or non‐native plants which may fail to support native wildlife (but see Harrison & Winfree, 2015; Padovani et al., 2020). Urban habitats place stronger environmental constraints on plant and animal communities than rural habitats (e.g. air pollution, noise and artificial light; Isaksson, 2015) and may disrupt ecological interactions between plants and pollinators via habitat fragmentation (Hennig & Ghazoul, 2011). Impacts on species richness in urban areas are dependent on the specific taxonomic group, the spatial scale of analysis and the intensity of urbanisation (Fournier et al., 2020; McKinney, 2008; Theodorou et al., 2020). A greater species richness in urban areas may be due to the increased number of both native and non‐native species, due to the larger species pools that urban areas maintain (Dolan et al., 2011), particularly when there are sufficient corridors of green space to allow colonisation from the regional species pool (Rega‐Brodsky et al., 2022). This could also be underpinned by the mosaic of habitat patches in urban ecosystems, and the associated heterogeneity of plant communities that could support biodiversity at higher trophic levels (Swan et al., 2021). Effects of urbanisation on top‐down control (e.g. altering predation by birds) or bottom‐up control (e.g. altering vegetation structure) could also lead to indirect effects on abundance, species diversity or community composition throughout the food web (Theodorou, 2022).

Urban areas can be characterised as a spatial assemblage of people whose lives are structured around non‐agricultural activities, with rural areas defined as any place that is not classified as urban (Weeks, 2010). The European Commission's classification system categorises areas along an urban–rural continuum, combining population size and density criteria to define cities, towns, semi‐dense regions and rural areas (Eurostat, 2019). Rapid urban development and expansion in recent years have altered many wildlife assemblages, especially invertebrates (Van Swaay & Warren, 1999). Perhaps the most well‐studied group is butterflies, as they are popular, easy to identify, and have been used as model insects for many years (Warren et al., 2021). But butterflies are also in decline due to severe habitat loss and climate change (Zografou et al., 2009). More generally, butterflies are important indicators of ecosystem health due to their susceptibility and sensitivity to changes in the environment (Ghazanfar et al., 2016). Butterflies have a high reproductive rate and occupy low trophic levels; thus, they respond quickly to environmental stressors and could be utilised as a proxy for general reductions in wildlife (Ghazanfar et al., 2016). Here, we focus on butterflies as indicator taxa, whilst considering the impact of urbanisation on their potential predators and resources.

Urbanisation has been shown to degrade bird communities through species decline and functional homogenisation (Tzortzakaki et al., 2018). The main factors affecting bird species assemblages are green space availability, noise pollution, interspecific competition and habitat heterogeneity (Chiron et al., 2024; Martin & Bonier, 2018; Rodrigues et al., 2018). Collisions with buildings in urban areas also heavily affects bird populations, including species of conservation concern (Hager et al., 2017). Vincze et al. (2017) found that in urbanised areas there was an increase in predation of bird nests by urban exploiters such as crows (*Corvus* spp.), magpies (*Pica pica* L., 1758) and cats (*Felis silvestris catus* L., 1758). However, it is also suggested that prey populations of birds thrive in urban areas as these habitats are low in abundance of larger predators (Vincze et al., 2017). Cities and towns have variability in terms of the activity or usage of areas, thus bird species distribution in urban areas is related to the degree of urbanisation and habitat features such as tree and shrub cover and the density of buildings (Rodrigues et al., 2018). Moreover, human landscape characteristics favour species that can exploit novel resources and adapt to new habitats, such as hooded crows (*Corvus cornix* L., 1758), house sparrows (*Passer domesticus* L., 1758) and pigeons (*Columbidae* spp.; Kark et al., 2007).

The high abundance of adaptive birds in urban environments could thus have negative impacts on invertebrates, specifically butterfly populations compared to rural habitats. For example, birds often achieve higher population densities in urban environments due to the lack of natural predators and abundance of food, which could lead to greater top‐down control on butterflies (Shochat et al., 2010). However, butterflies have developed various defensive traits against birds, such as chemical cues and aposematic or cryptic colouration, that is, bright colours in conspicuous patterns on the wings (Paladini et al., 2018). Additionally, many butterflies have adopted fast, unpredictable flight and weak, fragile wings that allow escape by tearing when pecked by birds (Pinheiro & Cintra, 2017). Brighter colouration signals are commonly associated with potent defence and greater reproductive success, as predators are naturally deterred, within‐species rivals are more cautious and potential mates are more interested (Yeager & Barnett, 2021). Due to the high frequency of beak marks on the wings of butterflies, birds are likely their most significant predator (Pinheiro & Cintra, 2017). Nonetheless, small mammals, toads and lizards also feed on adult butterflies, and there may be significant predation by a variety of invertebrates (Londt, 1999).

Changes in the patterns of vegetation composition and structure in urban areas, can lead to a reduction of bird species richness and selection for omnivores, carnivores, and species which nest in cavities (de Toledo et al., 2012). But native vegetation diversity within green spaces can strengthen the abundance and richness of specialist and insectivorous bird species (Silva et al., 2021). Plant biodiversity often increases in urban areas through the introduction of exotic (non‐native) species (Peng & Liu, 2007), but this is strongly dependent on the influence of human preferences and management activities (Avolio et al., 2021). The introduction of non‐native plant species in urban areas degrades habitats and shifts community composition, however, often with huge turnover of species across urban habitats, which can influence ecosystem services and habitat resilience (Dolen et al., 2011; Swan et al., 2021). Urbanisation also alters the timing of important reoccurring plant phenology events, such as flowering and leaf‐out, leading to cascading consequences on the species within a community and disturbing important interactions such as pollination and herbivory (Dale & Frank, 2018; Li et al., 2019). The gross primary productivity of vegetation also decreases with increasing levels of urbanisation from loss of green land and changing macro‐environment (Chen et al., 2022). While habitat enhancements of exotic species may increase ecosystem resilience and integrity, restoration of native communities in urban areas may increase connectivity to surrounding rural landscapes and support native ecosystems (de Carvalho et al., 2022).

There is a mutual and historical co‐evolution in operation between plants and invertebrates (Ghazanfar et al., 2016). Co‐evolutionary traits include adaptive radiation of plants that evolved to have chemical protection from herbivores, followed by adaptive radiation in herbivores who developed characteristics to counter this defence (Feeny, 1975). For example, the butterfly proboscis attachment has adapted to reach the nectar at the base of long‐tubed flowers (Ghazanfar et al., 2016). Alternatively, some skippers (Hesperiidae) are only capable of utilising shallow blossoms, such as flowers in the myrtle family (Myrtaceae; Ghazanfar et al., 2016). Increasing urbanisation results in fewer plant species visited, indicating lower resource use or availability for pollinators in urban environments (Ellis et al., 2023). Smaller plant patches found in urban environments tend to receive fewer pollinator visits and suffer pollen limitation (Barker, 2018). This reduces genetic exchange and flowering plant diversity, and consequently, supports fewer pollinator species. Yet, low building density and the presence of green space within urban areas, may drive pollinator movement and thus gene flow between patches (Hennig & Ghazoul, 2011).

Whilst anthropogenic disturbances are fostering negative impacts on butterfly species, human practices have created agricultural and woodland management systems such as hay meadows and coppicing that assist the growth of butterfly populations (Dover & Settele, 2009). The Mediterranean is one of the world's 25 biodiversity hotspots, mainly due to the abundance of endemic species within this area (Lopez‐Villalta, 2010). The Aegean Sea is located within the Mediterranean where butterfly species vary between the islands. In this area, Haahtela et al. (2019) recorded the highest levels of diversity on Samos Island (64 species) and Lesbos Island (63 species; Haahtela et al., 2019). The evolution, extinction and species migration of animals and plant species over archipelago islands are reflected in the pattern of species diversity (Dennis et al., 2000). Therefore, a distinct and endemic species assemblage of butterflies may be present across the Aegean islands. This highlights the importance of green space within Mediterranean urban areas and a demand to assess the butterfly species within this environment. The study of butterflies within the Aegean region is severely lacking and mainly focuses on biogeographical studies (e.g. Dennis et al., 2000; Hammoud et al., 2021; Hausdorf & Hennig, 2005), thus, the specific habitat types that butterflies utilise is not known. When studying Tuz Lake in Turkey, Seven (2017) compared habitat preferences of butterflies and observed the highest species diversity within the steppe habitat (defined as semi‐arid grassland) and the lowest diversity in poorly vegetated areas dominated by rocks, indicating that species may prefer vegetated and shaded areas. Due to the global decline of butterflies, the exploration of urban green space as a possible diversity hotspot is crucial and contributes to current research.

Increasing urbanisation due to ongoing development of islands in the Aegean region makes it essential to study the impacts of even low‐intensity urbanisation on butterfly communities and their potential predators and resources. This is particularly relevant given the paucity of research on butterfly ecology within the Aegean. Thus, a key novel contribution of this study is to compare the ecological communities found in rural areas and urban green spaces on Lipsi Island, Greece. It is hypothesised that total abundance, species richness and Shannon diversity of (1) butterflies, (2) birds, and (3) vegetation will be higher in rural compared to urban sites and that (4) urbanisation will have an impact on community composition of each trophic group.

## 2 METHODOLOGY

### 2.1 Study sites

The study was conducted during the months of May and June 2021 on Lipsi Island, Greece (approximate area: 17 km^2^), which is located within the eastern Aegean Sea (37°17′44.7″ N, 26°46′45.5″ E) and used as a model small island ecosystem. The town centre of Lipsi Island, with approximately 700 inhabitants in a 1 km^2^ area, falls within the scope of an intermediate density area, and can be referred to as a town or small urban zone (Eurostat, 2019). Thus, the island experiences low‐intensity urbanisation on a global scale (building density = 15–20% in urban areas) and so the ecological impacts of urbanisation on Lipsi should be distinguished from the typical literature on large urban areas. Nevertheless, the impacts of urban development on the natural landscape of small island ecosystems can be comparatively greater than in built‐up areas and warrant investigation (Fernandes & Pinho, 2017). Sampling over an entire year was not logistically feasible, so we chose this timescale because previous studies in Mediterranean regions indicated that peak butterfly activity and abundance should occur in May and June (Fileccia et al., 2015; Hantson & Baz, 2013). The lower abundance and richness observed in early spring and late summer is mainly due to a reduction in flower diversity and, thus nectar sources for pollinators (Hantson & Baz, 2013).

**FIGURE 1:**
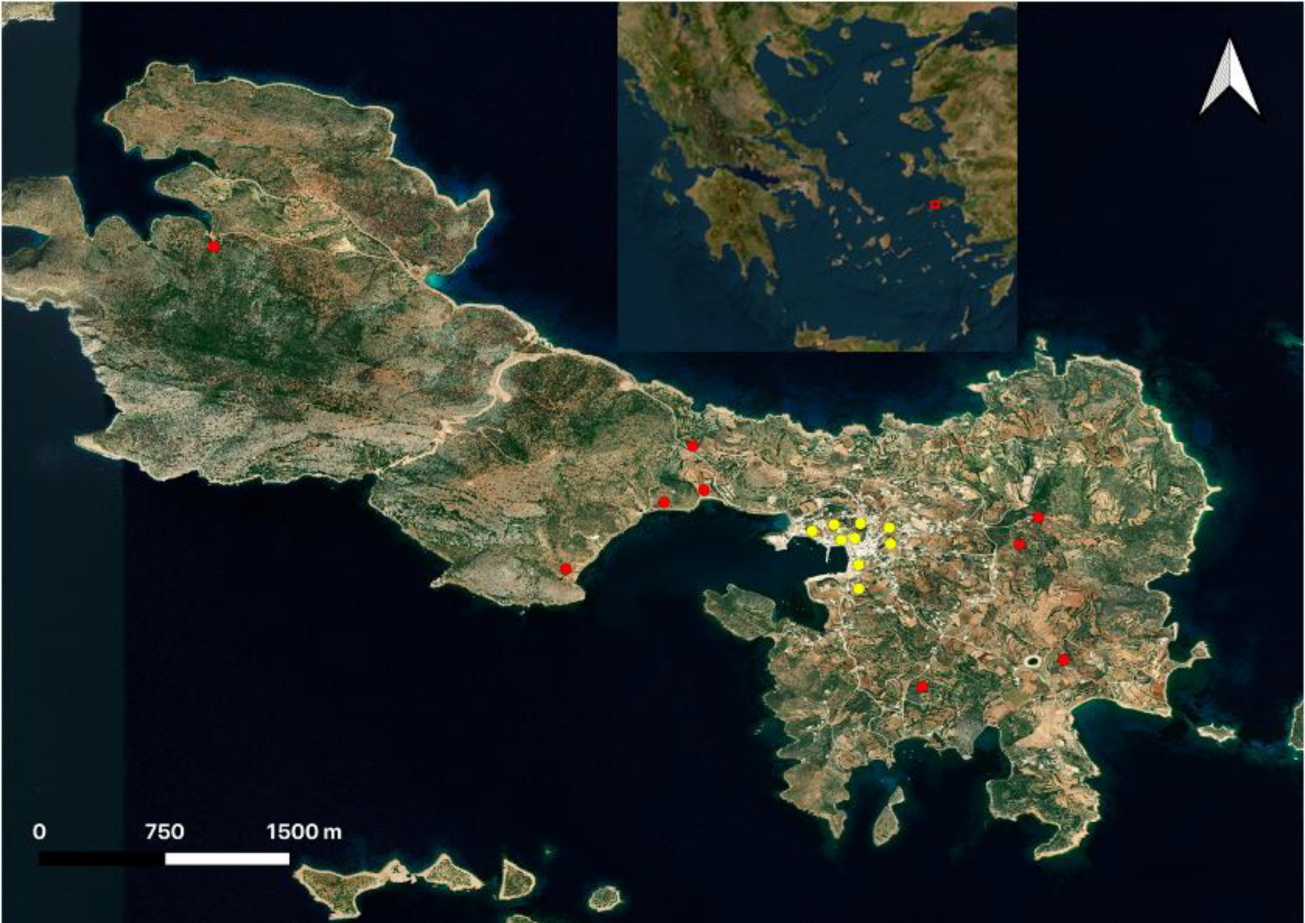
Study location. Map of Lipsi Island, Greece. The red square in the inst indicates the location of Lipsi Island in the Aegean Sea. Yellow dots indicate the nine urban sites and red dots indicate the nine rural sites (created in QGIS, satellite imagery from ESRI, 2011).

Nine urban and nine rural sites with clear separation were selected (Figure 1). The minimum distance between study sites in urban areas was 75.3 m, which limited the double counting of individuals. Due to the lack of trees, shade is restricted on Lipsi Island, thus, locations with high light intensity and low shade were utilised to give an accurate representation of the urban and rural habitats used by butterfly species. Sites were chosen to represent the predominant land‐use types utilised by butterflies during one or several stages of their life cycle (Grill & Cleary, 2003). The chosen rural habitats were shrubland, olive groves and meadows, while the urban habitats included agricultural meadows, abandoned land, parks, roadsides and olive groves. The sites were similar in size to keep the sampling effort consistent and included some shade to account for butterfly preference for shelter from the sun.

### 2.2 Butterfly sampling

**FIGURE 2:**
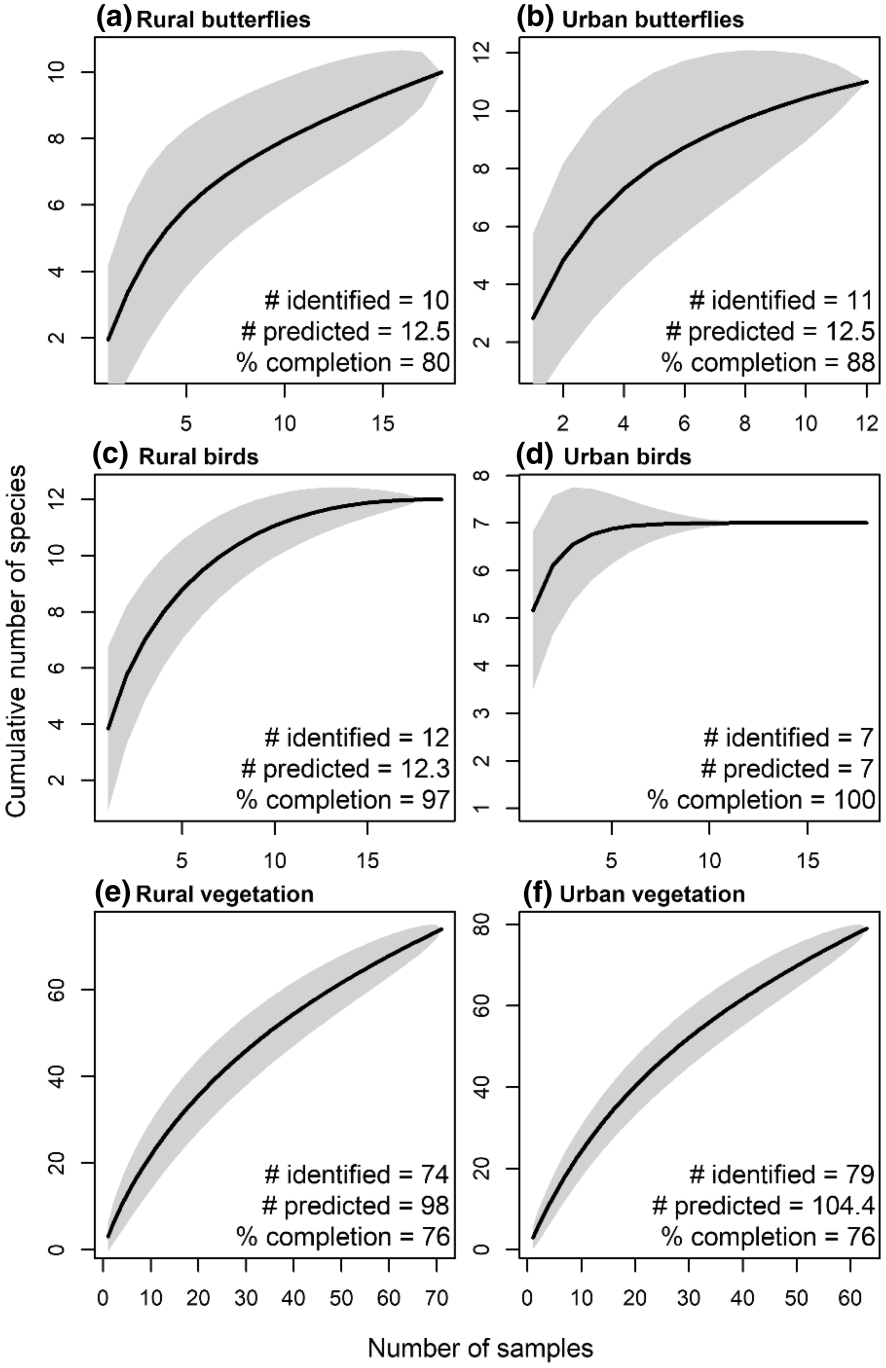
Species accumulation curves for documenting completeness of sampling. (a) Butterflies in rural habitats, (b) butterflies in urban habitats, (c) birds in rural habitats, (d) birds in urban habitats, (e) vegetation in rural habitats and (f) vegetation in urban habitats. The thick black line is the species accumulation curve estimated using the Mao Tao method with 95% confidence intervals shaded in grey. The number of species identified is shown, along with the number of species predicted for twice the number samples collected, and percentage completion of sampling.

The sampling technique implemented was the butterfly census method, which is widely used by the UK Butterfly Monitoring Scheme (UKBMS). Developed in 1973 by Ernest Pollard (Sevilleja et al., 2019), this method uses W‐ or M‐shaped transects to cover heterogeneity within the sampling area. The key variables to standardise with this method are the transect length, walking speed, time of day and weather (Wheater et al., 2011). The implemented method was adapted from Zografou et al. (2009). The four corners of each site were located using QGIS to create a square shaped plot for one Pollard transect, ranging from 40 to 70 m. The average site size for both urban and rural plots was 583.2 ± 55.1 m^2^ (mean ± SE). Butterflies observed 5 m in front and on either side of the transect were recorded and identified. According to Wheater et al. (2011), butterfly surveys should be performed between 10:00 and 16:00, but preliminary surveys indicated that butterflies on Lipsi were very sensitive to changes in temperature during these hours and the highest abundance of butterflies was found before 10:00. Therefore, butterfly surveys were undertaken between 7:00 and 10:00 with temperatures <27°C and wind conditions <25 km/h. The ‘*Butterflies of Britain and Europe: A photographic guide*’ was used for species identification (Haahtela et al., 2019). One transect was conducted at each site in both May and June for a total sample size of *n* = 36, which was sufficient to characterise >80% of the butterfly community at both rural and urban sites (Figure 2a,b). The incomplete nature of sampling indicates that butterfly results should be interpreted with some caution.

### 2.3 Bird sampling

Point‐counts were also implemented to quantify the degree to which birds affect butterfly populations in urban and rural habitats (Huff et al., 2000). Using binoculars, two people recorded species and number of individuals in point‐count surveys for 5 min in each compass direction, starting with North, and rotating through East, South and West. The total survey time was 20 min, whereby 5 min in each direction within a small sampling site helps to avoid counting the same individuals twice (Lee & Marsden, 2008). The start and end times were recorded, as well as the species and number of individuals. Bird distance to the habitat was also estimated and assigned one of the four categories: (1) 0–50 m from the station centre point: birds up to top of vegetation or canopy; (2) >50 m from the station centre point: birds up to top of vegetation or canopy; (3) Fly‐over associated with the habitat: birds above the top of the canopy and (4) Fly‐over independent of the habitats: birds above the top of the canopy, which do not seem to be interacting with the environment (Huff et al., 2000). Observations took place between 5:30 and 10:00 with wind <30 km/h and when there was no rain or fog. The ‘*Birds of Greece*’ was used for species identification (Nason, 2020). This sampling method was conducted at each site in May and June for a total sample size of *n* = 36, which was sufficient to characterise >98% of the bird community at both rural and urban sites (Figure 2c,d).

### 2.4 Vegetation sampling

On the first visit to each site, the percentage cover of shrubs and bare ground were recorded using the same quadrats for percentage cover of plants, and the percentage cover of trees was later recorded using Google Maps. Plant surveys were conducted following Tzortzakaki et al. (2019), with the app ‘PictureThis’ used alongside local taxonomic expertise for species identification (Glority Global Group Limited, 2020). Four 0.5 m^2^ quadrats were established at even distances along the butterfly transects, with the small quadrat size chosen due to the limited spatial extent of the sites. The percentage cover of each plant species was recorded for each quadrat, with vegetation surveys conducted at each site in May and June for a total sample size of *n* = 144. Despite the increased sample size compared to the butterflies and birds, species accumulation curves suggested that we only described 76% of the vegetation community in each habitat type (Figure 2e,f), thus vegetation results should be interpreted with caution.

### 2.5 Data analysis

The abundance, species richness, and species diversity of vegetation, butterflies and birds found at each site were quantified as the number of individuals, number of unique species and Shannon index respectively. Linear mixed effects models were performed on all response variables with a random intercept for sampling time point to account for the non‐independence of repeated sampling at the same location. No spatial structure was included in the models because there was no clear evidence for spatial correlation in the data using Moran's I tests. Non‐metric multidimensional scaling (NMDS) was used to explore the differences in community composition between rural and urban sites, with significant differences tested using PERMANOVA. All analyses were performed using R 4.0.2 (R Core Team, 2021). Data were organised using the ‘*tidyr*’ package (Wickham et al., 2019), graphs were created using ‘*ggplot2*’ (Wickham et al., 2019), ‘*cowplot*’ (Wilke, 2019) and ‘*gridExtra*’ (Auguie, 2017), and diversity and ordination analysis were performed with the ‘*vegan*’ package (Oksanen et al., 2019). Species accumulation curves were constructed using the ‘*specaccum*’ function with ‘*method = “exact”*’, while the predicted number of species per habitat type was estimated using the ‘*fitspecaccum*’ function and AIC selection among the nine possible non‐linear regression models available within the function in the ‘*vegan*’ package. Rank‐abundance plots were constructed using the ‘*rankabundance*’ and ‘*rankabunplot*’ functions in the ‘*BiodiversityR*’ package. Statistical analyses were conducted using the ‘*lme*’ function in the ‘*nlme*’ package and the ‘*adonis2*’ function in the ‘*vegan*’ package.

## 3 RESULTS

**FIGURE 3:**
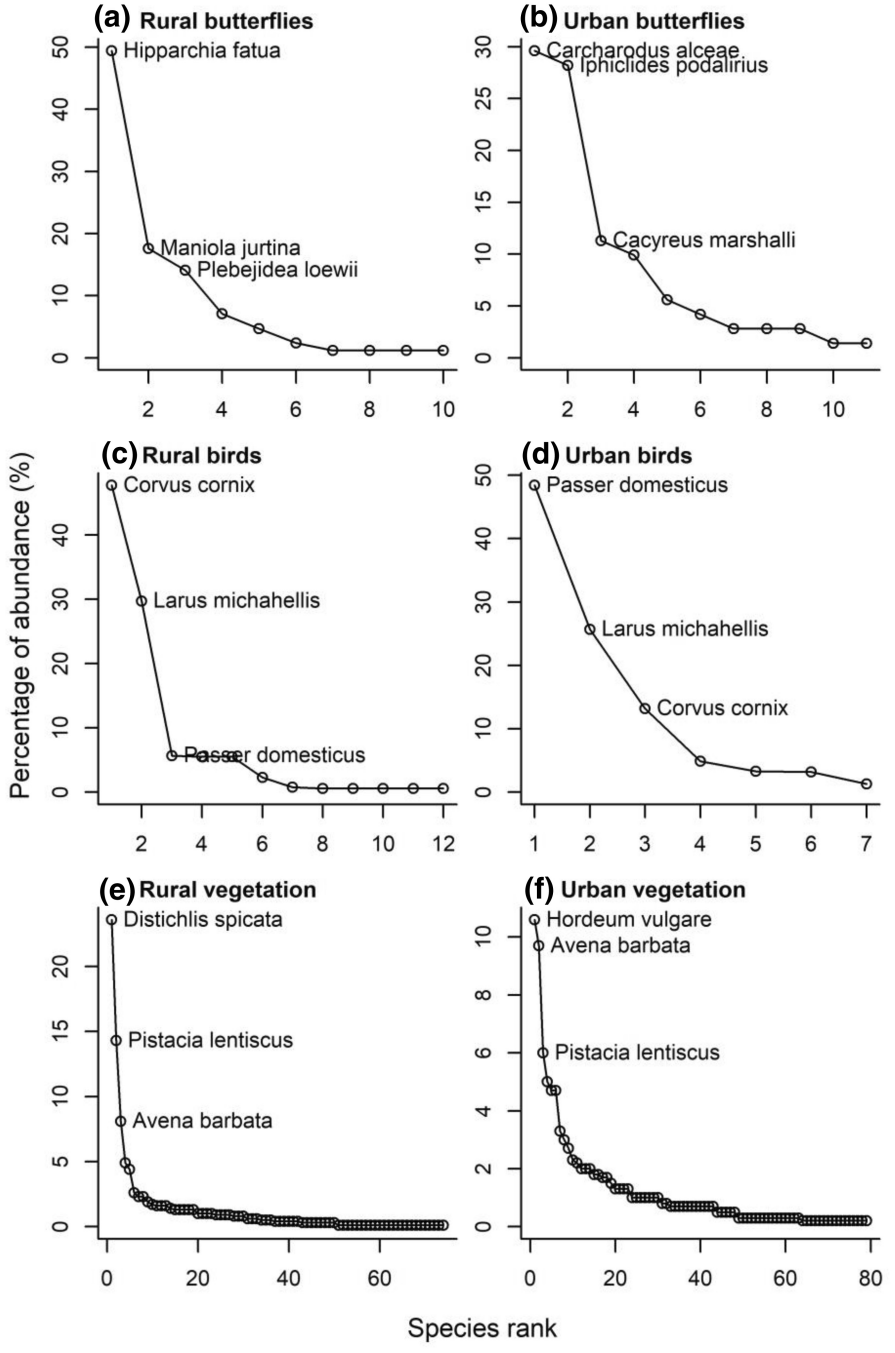
Rank‐abundance plots showing the three most dominant species from each trophic group in each habitat type. (a) Butterflies in rural habitats, (b) butterflies in urban habitats, (c) birds in rural habitats, (d) birds in urban habitats, (e) vegetation in rural habitats and (f) vegetation in urban habitats.

A total of 156 butterfly individuals (85 rural and 71 urban) from 14 species, 1668 bird individuals (511 rural and 1157 urban) from 12 species and a 15 ± 0.9 (mean ± SE) percentage cover of plants from 115 species (220 rural and 189 urban) were recorded across the 18 study sites. The three most abundant butterfly species in rural sites were Freyer's grayling (*Hipparchia fatua* Freyer, 1844; 42 in rural, 7 in urban), meadow brown (*Maniola jurtina* L., 1758; 15 in rural, 2 in urban) and the large jewel blue (*Plebejidea loewii* Zelter, 1847; 12 in rural; 3 in urban; Figure 3a). The three most abundant butterfly species in urban sites were the mallow skipper (*Carcharodus alceae* Esper, 1780; 21 in urban; 0 in rural), scarce swallowtail (*Iphiclides podalirius* L., 1758; 20 in urban, 1 in rural) and geranium bronze (*Cacyreus marshalli* Butler, 1898; 8 in urban; 0 in rural; Figure 3b). The most abundant bird species in rural sites was the hooded crow (*Corvus cornix* L., 1758; 243 in rural, 152 in urban; Figure 3c), the most abundant bird species in urban sites was the house sparrow (*Passer domesticus* L., 1758; 559 in urban, 29 in rural; Figure 3d), while the yellow‐legged gull was also dominant in both habitat types (*Larus michahellis* Naumann, 1840; 277 in urban, 152 in rural; Figure 3c,d). The most abundant vegetation species in rural sites was desert saltgrass (*Distichlis spicata* Greene, 1887; covering 23.6% in rural sites; 1.0% in urban; Figure 3e), the most abundant vegetation species in urban sites was barley (*Hordeum vulgare* L., 1753; covering 10.6% in urban sites; 0.9% in rural; Figure 3f), while mastic shrub (*Pistacia lentiscus* L., 1753; covering 14.3% in rural sites; 6.0% in urban) and slender wild oat (*Avena barbata* Link, 1799; covering 8.1% in rural sites; 9.7% in urban) were also dominant in both habitat types (Figure 3e,f).

### 3.1 Butterflies

**FIGURE 4:**
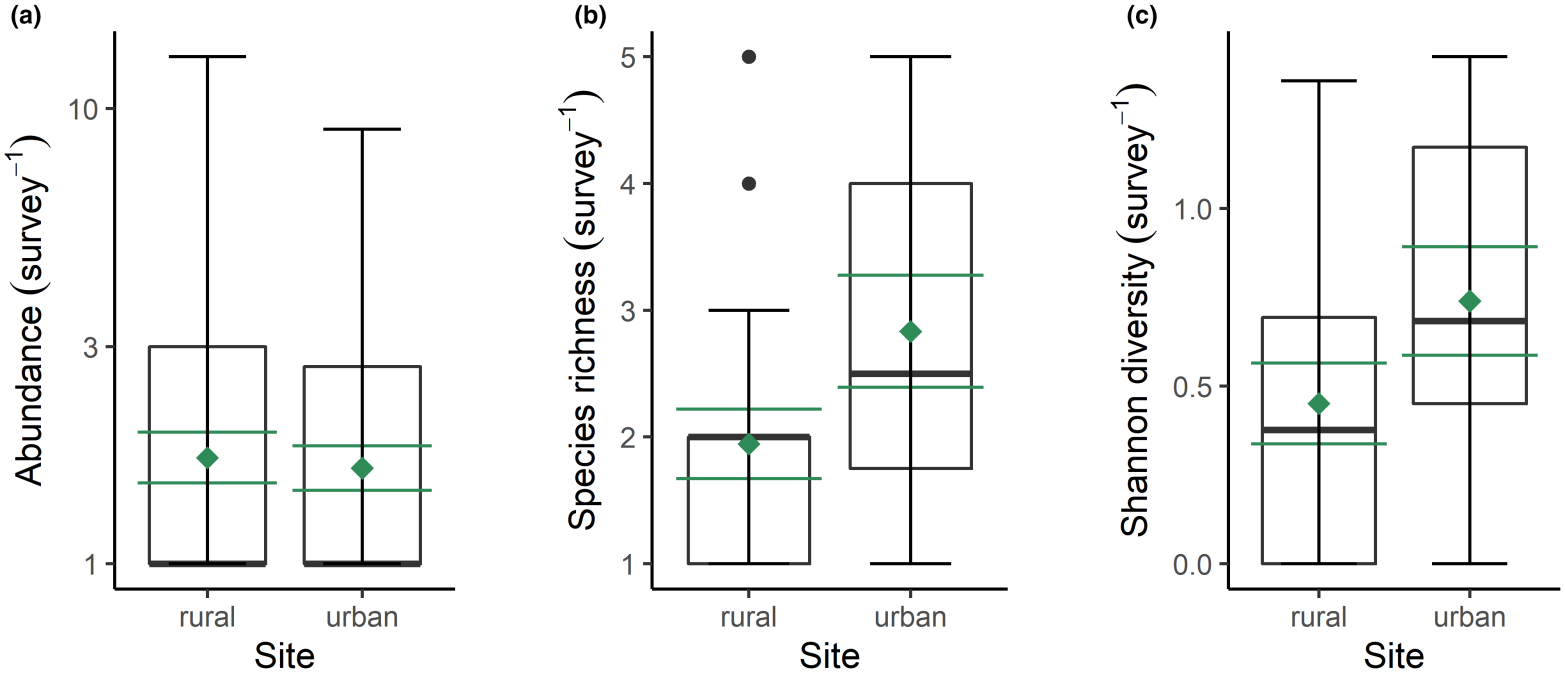
Effects of urbanisation on butterflies. (a) Abundance, (b) species richness and (c) Shannon diversity of butterflies at nine urban and nine rural sites. The black boxplots illustrate the median (bold black line), interquartile range (box margins), 1.5 × interquartile range (whiskers) and outliers (black data points), whilst the mean ± SE are represented by the green diamond and whiskers.

The abundance of butterflies was greater at rural (2.43 ± 0.44; mean ± SE) compared to urban sites (2.09 ± 0.31), but there was no significant difference between the two locations (Linear mixed effects model: *t* = −0.71, *p* = .480; Figure 4a). The species richness of butterflies was greater at urban (2.83 ± 0.44) compared to rural sites (1.94 ± 0.27), but there was no significant difference between locations (Linear mixed effects model: *t* = 1.81, *p* = .081; Figure 4b). The Shannon diversity of butterflies was also greater at urban (0.74 ± 0.15) compared to rural sites (0.45 ± 0.11), however the two locations did not differ significantly (Linear mixed effects model: *t* = 1.54, *p* = .135; Figure 4c). These results do not support our first hypothesis.

### 3.2 Birds

**FIGURE 5:**
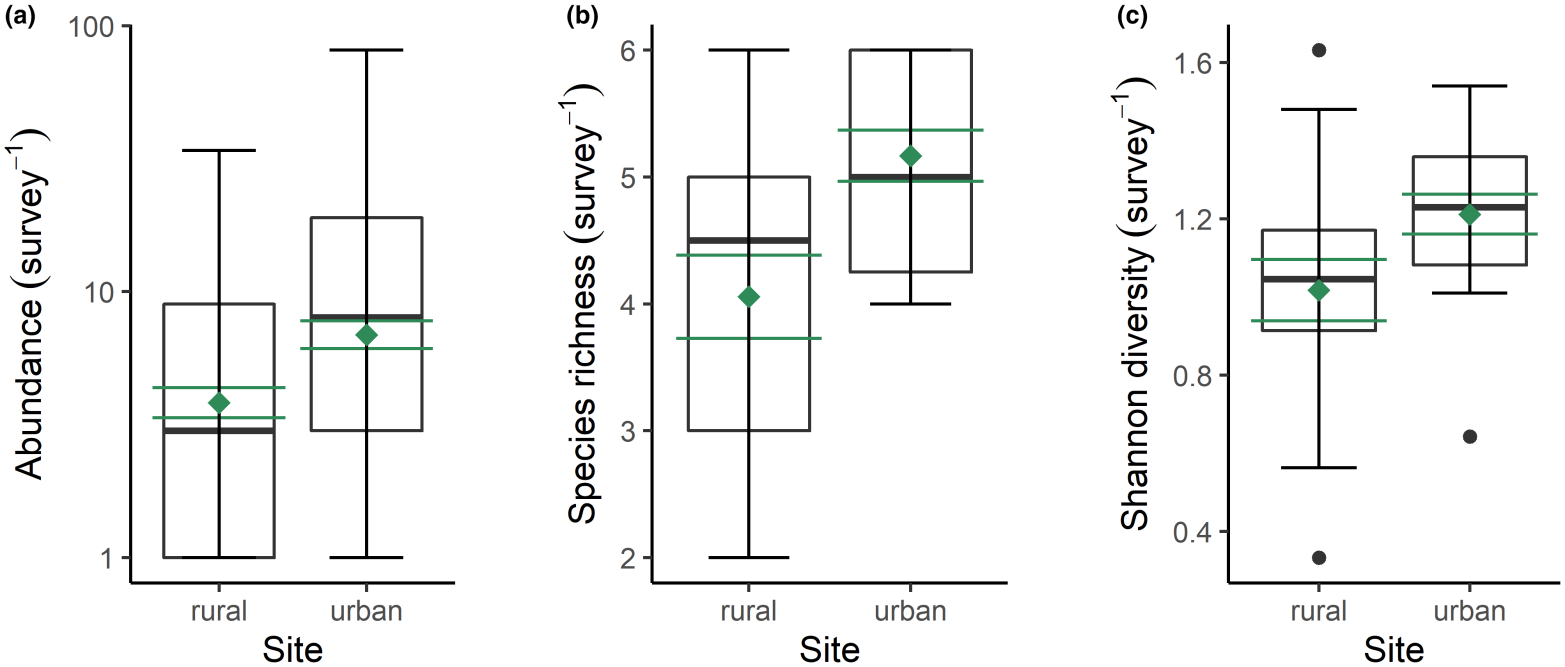
Effects of urbanisation on birds. (a) Abundance, (b) species richness and (c) Shannon diversity of birds at nine urban and nine rural sites. The black boxplots illustrate the median (bold black line), interquartile range (box margins), 1.5 × interquartile range (whiskers) and outliers (black data points), whilst the mean ± SE are represented by the green diamond and whiskers.

The abundance of birds was greater at urban (6.06 ± 0.56; mean ± SE) compared to rural sites (4.56 ± 0.55), and there was a significant difference between the two locations (Linear mixed effects model: *t* = 2.98, *p* = .003; Figure 5a). The species richness of birds was greater at urban (2.85 ± 0.18) compared to rural sites (2.19 ± 0.18), and both locations differed significantly (Linear mixed effects model: *t* = 2.88, *p* = .007; Figure 5b). The species diversity of birds was greater at urban (0.67 ± 0.06) compared to rural sites (0.51 ± 0.07), and there was a significant difference between the two locations (Linear mixed effects model: *t* = 2.07, *p* = .046; Figure 5c). These results are directly opposite to our second hypothesis, with evidence for a greater abundance, species richness, and diversity of birds in urban, not rural habitats.

### 3.3 Vegetation

**FIGURE 6:**
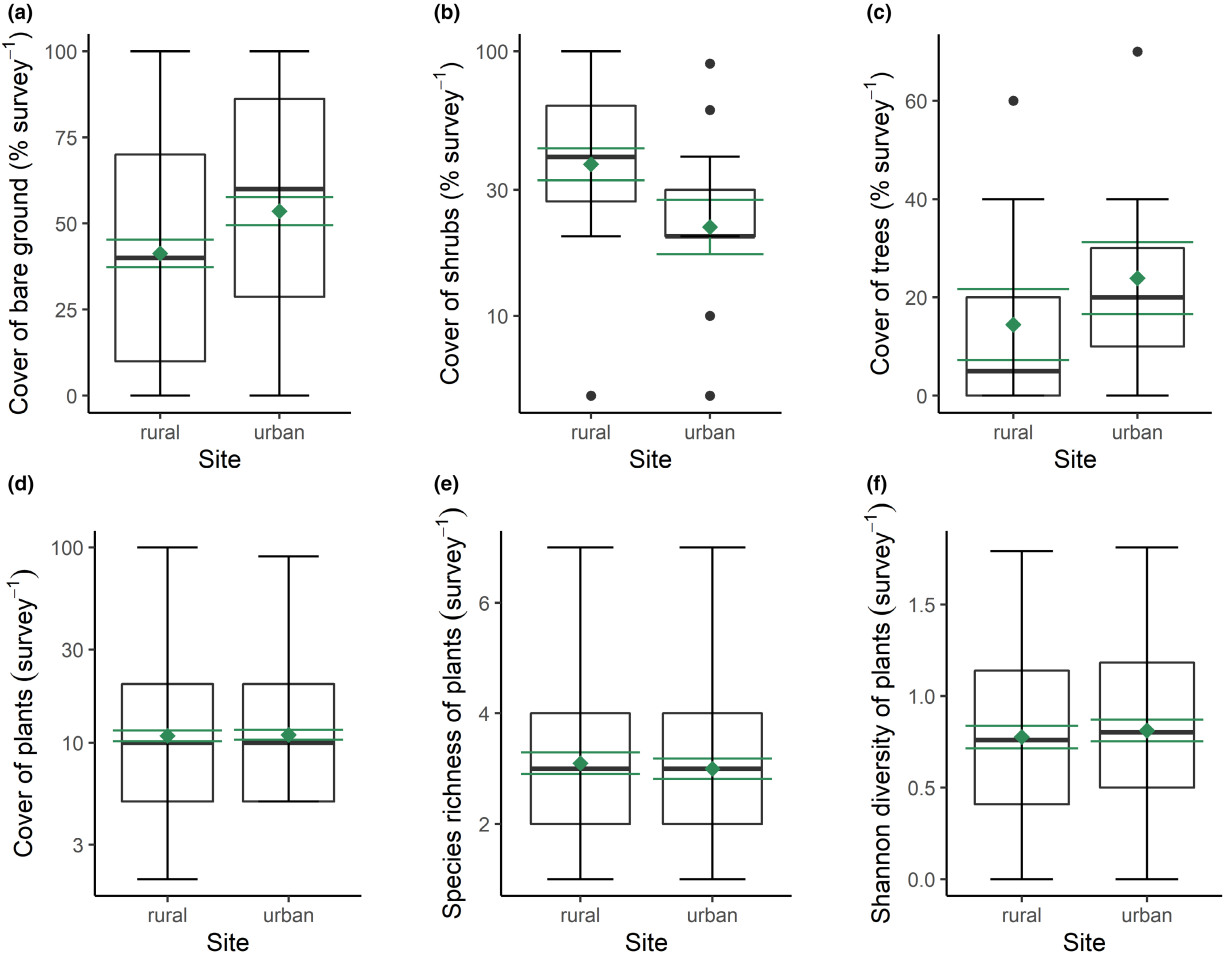
Effects of urbanisation on vegetation. Percentage cover of (a) bare ground, (b) shrubs, (c) trees and (d) plants, (e) plant species richness and (f) Shannon diversity of plants at nine urban and nine rural sites. The black boxplots illustrate the median (bold black line), interquartile range (box margins), 1.5 × interquartile range (whiskers) and outliers (black data points), whilst the mean ± SE are represented by the green diamond and whiskers.

The percentage cover of bare ground was greater at urban (53.6 ± 20.0; mean ± SE) compared to rural (41.3 ± 26.6) sites, and there was a significant difference between the two locations (Linear mixed effects model: *t* = 2.18, *p* = .032; Figure 6a). The percentage cover of shrubs was lower at urban (5.28 ± 7.41) compared to rural (15.1 ± 14.2) sites, and both locations differed significantly (Linear mixed effects model: *t* = −2.76, *p* = .007; Figure 6b). The percentage cover of trees was greater in urban (23.9 ± 22.0) compared to rural sites (14.4 ± 21.7), but the two locations were not significantly different (Linear mixed effects model: *t* = 0.916, *p* = .374; Figure 6c). The percentage cover of plants was greater at rural (17.5 ± 1.31) compared to urban (15.9 ± 1.24) sites, but there was no significant difference between the two locations (Linear mixed effects model: *t* = −0.365, *p* = .716; Figure 6d). The species richness of vegetation was greater at rural (3.10 ± 0.19) compared to urban sites (3.00 ± 0.18), but there was no significant difference between locations (Linear mixed effects model: *t* = −0.365, *p* = .716; Figure 6e). The Shannon diversity of vegetation was greater at urban (0.81 ± 0.06) compared to rural sites (0.77 ± 0.06), but the two locations were not significantly different (Linear mixed effects model: *t* = 0.420, *p* = .675; Figure 6f). These results do not support our third hypothesis.

### 3.4 Community composition

**FIGURE 7:**
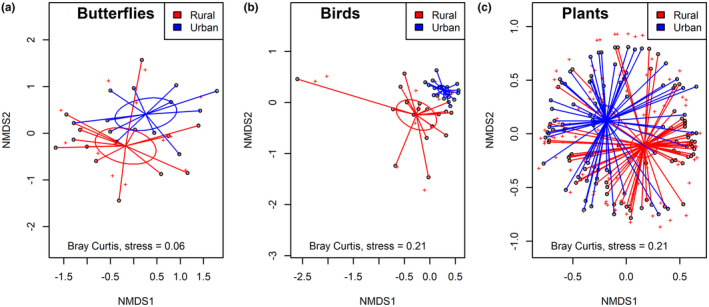
Effects of urbanisation on community composition. Non‐metric multidimensional scaling of (a) butterfly, (b) bird and (c) vegetation community composition. The black circles represent the sites, the red crosses indicate the species, and ellipses indicate the standard deviation of urban (blue) and rural (red) sites. The stress indicates the reliability of the points in two dimensions (lower values of stress equate to higher confidence).

There was a significant effect of urbanisation on butterfly community composition (PERMANOVA: *F*_1,28_ = 3.34; *p* < .001), with a clear separation between urban and rural sites in NMDS space (Figure 7a). There was also a significant effect of urbanisation on bird community composition (PERMANOVA: *F*_1,34_ = 15.57, *p* < .001), with a clear separation between rural and urban sites in NMDS space (Figure 7b). Finally, there was a significant effect of urbanisation on vegetation community composition (PERMANOVA: *F*_1,132_ = 4.81, *p* < .001), with a clear separation between urban and rural sites in NMDS space (Figure 7c). These results conclusively support our fourth hypothesis.

## 4 DISCUSSION

### 4.1 Butterflies

Surprisingly, urbanisation had no effect on the abundance, richness or diversity of butterfly species on Lipsi Island (Figure 4). Habitat connectivity may be amplified due to the small spatial extent of the island, enabling butterflies to utilise both rural and urban habitats. The low building density and development of ecological corridors on Lipsi may also increase movement between urban and rural areas (Hennig & Ghazoul, 2011). Alternatively, the lack of response could be due to incomplete sampling of butterflies, with species accumulation curves indicating that 80–88% of potential butterfly species were captured. Sample sizes were also low with nine sites in each habitat type, which may have inflated the possibility of Type 2 errors, that is, false negatives. Limiting our sampling to 2 months in the summertime may also have reduced our scope to detect potential effects of urbanisation on butterfly abundance and diversity. It would be intriguing to monitor seasonal patterns of butterflies over the course of an entire year, but financial and logistical constraints meant we chose to conduct this study during peak butterfly activity in May and June 2021. Whilst this likely means we did not characterise the entire communities that can be found throughout the year, it gave us an indication of their abundance, diversity, and community composition in the peak season.

Nonetheless, there was a significant change in butterfly community composition between urban and rural habitats (Figure 7a), as found in several other locations (Numa et al., 2016; Stefanescu et al., 2004; Tzortzakaki et al., 2019). For example, Freyer's grayling (*H. fatua*) was the dominant butterfly in rural areas but was comparatively rare in urban settings (Figure 2a,b), reflecting its preference for meadows and thus a lack of suitable habitat in urban areas (Grill & Cleary, 2003). In contrast, the scarce swallowtail (*I. podalirius*) was mainly found in urban environments (Figure 2a,b), reflecting its tendency to feed exclusively on rose plants, which are more likely to be found in urban gardens (Stefanescu et al., 2006). Butterfly species respond differently to the environmental constraints encountered along an urbanisation gradient due to variation in tolerance levels associated with life history and distribution (Pignataro et al., 2020). For example, a study in Patras city, Greece, showed that specialist butterfly species with specific feeding requirements were often absent from urban environments, whereas generalists exhibited a greater abundance in urban areas (Tzortzakaki et al., 2019). Habitat fragmentation and reduced connectivity due to urbanisation may lead to a decline in specialist species within these areas (Brückmann et al., 2010; Kuussaari et al., 2021), however, the geranium bronze (*C. marshalli*) and mallow skipper (*C. alceae*) were more abundant in urban areas on Lipsi, despite being specialists. Geranium bronze is highly associated with cultivated geranium plants (*Pelargonium*) found in gardens and parks, whilst the mallow skipper caterpillar feeds on mallow plants (Malvaceae) which are weeds found in urban waste ground, roadsides and gardens (Tzortzakaki et al., 2019). Therefore, the presence of cultivated plants within urban locations could mitigate the loss of natural vegetation and support certain specialist species (Chong et al., 2014), whilst generalist or opportunistic butterflies may be able to exploit the resources found in both urban and rural locations (Pignataro et al., 2020).

### 4.2 Birds

Urban sites had a greater abundance and species richness of birds compared to rural sites (Figure 5), which could be attributed to the addition of species accustomed to urban environments, termed ‘urban exploiters’ (Crooks et al., 2004). For example, rock doves (*Columba livia* Gmelin, 1789) and house sparrows (*P. domesticus*) are dexterous at exploiting discarded food, utilising human made nesting sites (roofs) and other resources in urban environments, and consequently achieving higher densities in developed areas (Blair, 1996). Indeed, sparrows were abundant in urban locations in our study (559 individuals) and were much less common in rural areas (29 individuals), echoing the finding of Belinsky et al. (2019). This dominance of adaptable bird species in urban locations underpinned the disparity in community composition compared to rural habitats (Figure 7b), with urban areas usually supporting fewer species from ecologically sensitive groups, for example, ground nesters, migratory birds and dietary specialists (Blair, 1996; Dale, 2018). Our findings differ from prior research suggesting that species richness is lower in urban areas due to the prevalence of buildings over vegetation (Kark et al., 2007; Tzortzakaki et al., 2018). Several studies have found that species richness peaks with intermediate levels of urbanisation which resonates with the low‐intensity urbanisation found on Lipsi Island (Blair, 1996; Crooks et al., 2004). Indeed, eight of our nine urban sites are naturally vegetated and underdeveloped, consistent with the findings from White et al. (2005), showing that underdeveloped areas had a greater abundance and species richness of birds compared to recently developed locations.

Nevertheless, the greater abundance and species richness of birds in urban areas did not seem to elevate the predation pressure on butterflies, which had similar abundance and diversity in urban and rural environments. This could be due to increased dominance of non‐predatory functional groups of birds, with Nason et al. (2021) finding a greater abundance of granivorous and omnivorous birds rather than insectivores in urban areas, perhaps due to the abundance of discarded food available there, causing a decline in overall bird attacks on animal prey. Indeed, urban dominance by omnivorous house sparrows, hooded crows and yellow‐legged gulls on Lipsi may have reduced predation pressure on butterflies in urban areas. However, the insectivorous barn swallows and common house martins were more abundant in urban (93 individuals) compared to rural (32 individuals) sites, suggesting complex effects of urbanisation on food web interactions that would require dietary studies to disentangle.

Changes in predation pressure are further complicated by responses of non‐avian predators to urbanisation. For example, robber flies (Asilidae spp), which are important predators of butterflies (Lehr et al., 2007; Londt, 1999), were seen in almost every shrubland site, but rarely in urban areas. Aposematism defence (use of vibrant colours) acts as a warning for predators (Pinheiro & Cintra, 2017), and butterflies with intricate camouflage such as meadow brown and graylings were observed to be more abundant in rural habitats (63 individuals) compared to urban locations (13 individuals). This may point to the greater predation pressure experienced by butterflies in rural areas and could help to explain the surprising similarity in abundance and diversity of butterflies in rural compared to urban environments.

### 4.3 Vegetation

The lack of significant differences in the abundance, richness and diversity of plants between urban and rural sites (Figure 6d–f) could be due to incomplete sampling, with just 76% of potential plant species identified. Nevertheless, our species accumulation curves exhibited long tails of rare plants, suggesting that any missed species would have contributed very little to the overall percentage cover of the plant communities. Indeed, two of the top three plants by percentage cover in both rural and urban environments were mastic shrub (*P. lentiscus*) and slender wild oat (*A. barbata*), highlighting the prevalence of native, unmanaged vegetation within urban green spaces on Lipsi. Whilst rural areas had significantly more shrubs and less bare ground than urban sites, there was no difference in the cover of trees, which are scarce on Lipsi, negating the possibility for greater tree cover to promote butterfly species richness (Kurylo et al., 2020). The lack of a marked difference in vegetation structure between urban and rural areas could thus be a key factor in explaining the similarity in butterfly abundance and diversity across habitats. A prevalence of non‐native plant species in urban areas may prevent larval development of butterflies (Dylewski et al., 2019), but exotic species were only present at three urban sites and in low abundance, limiting their potentially negative effects on butterflies. Furthermore, butterfly abundance responds negatively to non‐native plants in late spring and positively by mid‐summer (Kurylo et al., 2020), highlighting the importance of greater temporal resolution of sampling to characterise vegetation effects on butterflies.

Urbanisation altered vegetation community composition (Figure 7c), with cultivated species such as barley (*H. vulgare*), castor bean (*Ricinus communis* L., 1753) and scutch grass (*Cynodon dactylon* Persoon, 1805) prevalent in urban environments, while natural desert saltgrass (*D. spicata*) dominated in rural environments. This may have contributed to the observed differences in butterfly community composition, with cultivated patches hosting different butterfly assemblages than natural forests and scrub (Chong et al., 2014). As noted above, the dominant butterflies in urban environments (geranium bronze, scarce swallowtail, and mallow skipper) are highly associated with cultivated plants, which were prevalent in urban areas (Stefanescu et al., 2006; Tzortzakaki et al., 2019). In contrast, the dominant butterflies in rural environments (Freyer's grayling and meadow brown) depend on meadows and grasslands, which were largely absent from urban areas (Grill & Cleary, 2003; Merckx & Van Dyck, 2002). Thus, whilst we did not detect any effect of urbanisation on the abundance or diversity of the vegetation and butterfly assemblages, the observed changes in dominance patterns and species composition between rural and urban environments could have major implications for ecosystem functioning, which should be quantified in future studies.

## 5 CONCLUSION

There remains a pressing concern of global declining butterfly populations, mainly due to anthropogenic pressure from urban development (Van Swaay & Warren, 1999). Green spaces within urban locations may help to maintain total abundance and species diversity (Hennig & Ghazoul, 2011), but habitat fragmentation and smaller size and quality of habitat patches will alter community composition (Belinsky et al., 2019). The structural similarities between urban and rural habitats and the very low intensity of urbanisation on Lipsi Island compared to many other urbanisation studies may have driven the overlap in abundance and diversity of butterflies and vegetation, whilst the greater habitat heterogeneity and discarded food in urban environments could have promoted the abundance and species richness of generalist and opportunistic birds. Thus, metrics other than simple counts of individuals and species are needed to characterise the impacts of urbanisation on community composition across multiple trophic levels. Future research should aim to characterise changes in community structure along a gradient of urban development and island size and the implications for ecosystem functioning. Whilst butterflies were considered as important indicator species here, follow‐up studies should quantify effects of urbanisation on other pollinators and arthropod assemblages for a more complete understanding of changes throughout the food web. Finally, dietary characterisation is required to quantify changes in the strength and diversity of ecological interactions, which could help elucidate impacts of urbanisation on the flow of energy through ecological networks.

## AUTHOR CONTRIBUTIONS

**Eleanor R. Hawkins:** Conceptualization (lead); data curation (lead); formal analysis (lead); investigation (lead); methodology (lead); project administration (equal); software (lead); validation (lead); visualization (lead); writing – original draft (lead); writing – review and editing (equal). **Laura Macrina:** Conceptualization (supporting); data curation (supporting); funding acquisition (equal); investigation (supporting); methodology (supporting); project administration (equal); resources (equal); software (supporting); supervision (equal); writing – review and editing (equal). **Alice Malcolm‐McKay:** Conceptualization (supporting); data curation (supporting); resources (equal); supervision (lead); writing – review and editing (equal). **Anastasia Miliou:** Funding acquisition (lead); project administration (equal); resources (lead); supervision (supporting); writing – review and editing (supporting). **Nicola Cotterill:** Conceptualization (equal); investigation (supporting); methodology (supporting); writing – review and editing (equal). **Eoin J. O'Gorman:** Conceptualization (supporting); data curation (supporting); formal analysis (supporting); investigation (supporting); methodology (equal); project administration (equal); resources (equal); software (supporting); supervision (lead); validation (equal); visualization (equal); writing – original draft (supporting); writing – review and editing (equal).

## CONFLICT OF INTEREST STATEMENT

The authors declare that they have no known competing financial interests or personal relationships that could have appeared to influence the work reported in this paper.

## Data Availability

The data that supports this study are available in Supporting Information.
